# Giant Incisional Hernia Repair Using Open Intraperitoneal Dual Mesh

**DOI:** 10.7759/cureus.27126

**Published:** 2022-07-21

**Authors:** Ercan Korkut, Nurhak Aksungur, Necip Altundaş, Salih Kara, Rıfat Peksöz, Gürkan Öztürk

**Affiliations:** 1 General Surgery, Atatürk University Faculty of Medicine, Erzurum, TUR; 2 General Surgery, Atatürk University Research Hospital, Erzurum, TUR

**Keywords:** open intraperitoneal dual mesh placement, recurrence, open onlay mesh, intraperitoneal dual mesh, ventral hernia, open incisional hernia repair

## Abstract

Aim

Giant incisional herniae are larger than 15 cm and are typically treated with an open approach. Our aim was to highlight the outcomes of treating giant incisional hernia using open intraperitoneal dual mesh.

Methods

Between January 2015 and December 2021, 25 patients with giant incisional hernias, where fascial defects were 15-30 cm, were evaluated retrospectively. Intraperitoneal dual mesh was used in all patients. The patients were evaluated in terms of age, gender, body mass index (BMI), previous abdominal surgeries, defect diameter, anesthesia method, length of hospital stay, drain application, complications, and recurrence.

Results

Eleven of the patients were male and 14 were female. The mean age was 62±13.5 years (29-82 years). The average BMI was 32 kg/m2 (20-52 kg/m2). The mean size of the fascial defect was 22±5.5 cm (15-30). The mean operation time was 90 minutes (70-130 minutes). Six patients had type I and II complications according to the Clavien-Dindo classification, specifically superficial skin infections, skin erosion, subcutaneous bleeding, and temporary ileus due to intestinal adhesion. During the average follow-up period of 36 months (6-70 months), no major complications were observed related to the recurrence and use of dual mesh.

Conclusion

In the treatment of giant incisional hernia, open intraperitoneal dual mesh application should be kept in mind as an effective treatment option with low complication and recurrence rates.

## Introduction

Incisional hernias are the most common long-term complication after abdominal surgery, with a rate of 9-12.5% in the first year, and up to 30% with increasing years [1-3].

The causes of incisional hernia are multifactorial and may occur due to patient factors and technical reasons. The pathophysiology lies in excessive tension in the incision and poor wound healing. The most common predisposing factors are obesity and postoperative surgical site infection. Wound infection increases the risk of developing an incisional hernia, incisional hernia develops in 20-25% of patients with wound infection. In patients with BMI >30 kg/m2, the risk of incisional hernia rises up to 30%. Other factors contributing to incisional hernia include local and systemic causes that impair wound healing, advanced age, pulmonary diseases, metabolic diseases, diabetes mellitus, immunosuppression and corticosteroid drug use, oncological causes, postoperative chemotherapy and radiotherapy, using the same incision in multiple surgeries, inadequate surgical technique. In addition, in patients at risk of developing abdominal compartment syndrome such as abdominal infection and intestinal edema, leaving the fascia open causes incisional hernia [1,4,5].

The use of mesh has become standard for herniae larger than 2 cm because of high recurrence rates [6-7]. Many different meshes with different properties have been introduced to the market for clinical use. Meshes are basically divided into two groups: biological and synthetic mesh. Biological meshes are produced from collagen-rich acellular tissues obtained from cadavers and animals. Polypropylene, polyester, and expanded polytetrafluoroethylene (ePTFE) meshes are the most commonly used synthetic meshes. Many different new meshes with weave properties and material differences are being developed [8-9].

Dual meshes are mainly developed from synthetic meshes. The intraperitoneal surface of the dual meshes is coated with a substance that prevents the intestines from adhering to the mesh. Its use is mandatory in laparoscopic repairs where the mesh is placed in the abdomen, as well as in cases where it is impossible to bring the tissues closer together, such as cases where large hernias are repaired with the open method [4,10].

Onlay, inlay, sublay-retromuscular, sublay-preperitoneal, and sublay-intraperitoneal techniques have been defined in relation to mesh application in the treatment of hernia by the European Hernia Society [3,11]. However, each method has its own advantages, technical difficulties, and complications. There is no consensus among surgeons on the optimal location for mesh placement and what type of mesh to use in incisional hernia repair [11,12].

Complications can be seen in the early and late periods after incisional hernia surgery. In the early period, perioperative bowel injury, seroma, bleeding, superficial or deep wound infection, mesh infection, skin necrosis, and mesh detachment due to inadequate fixation of the mesh can be seen. Late complications are hernia recurrence and mesh reaction [11,12].

Our aim was to highlight the outcomes of treating giant incisional hernia using intraperitoneal dual mesh.

## Materials and methods

The approval for the study was obtained from the Ethics Committee of Erzurum Atatürk University Faculty of Medicine (No: B.30.2.ATA.0.01.00/464).

Patients who were operated on for incisional hernias between January 2015 and December 2021 were retrospectively investigated. Twenty-five patients with fascial defects between 15 cm and 30 cm and operated on using open intraperitoneal dual mesh were included in the study. The fascial defect was not suitable for primary closure in any of the patients. All patients were evaluated with abdominal CT. Age, gender, procedural time, body mass index (BMI), postoperative analgesic use, hospital stay, postoperative pain, complications, and recurrences were recorded. A dual mesh containing intraperitoneal-sided hydrogel barrier polypropylene mesh was used. Intravenous 1 g cefazolin sodium was administered to all patients before anesthesia induction.

Surgical technique intraperitoneal mesh application

**Figure 1:**
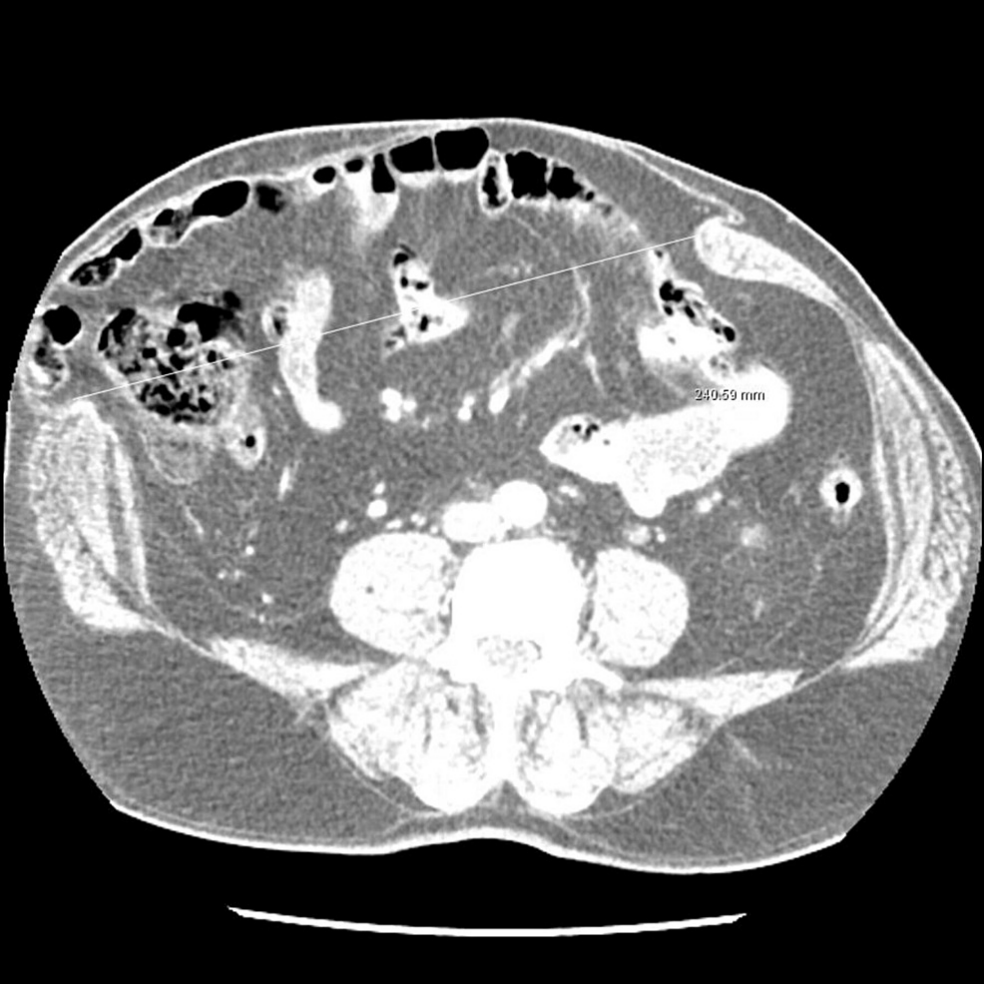
Axial section CT image of the sample patient with a fascial defect of approximately 24 cm

**Figure 2:**
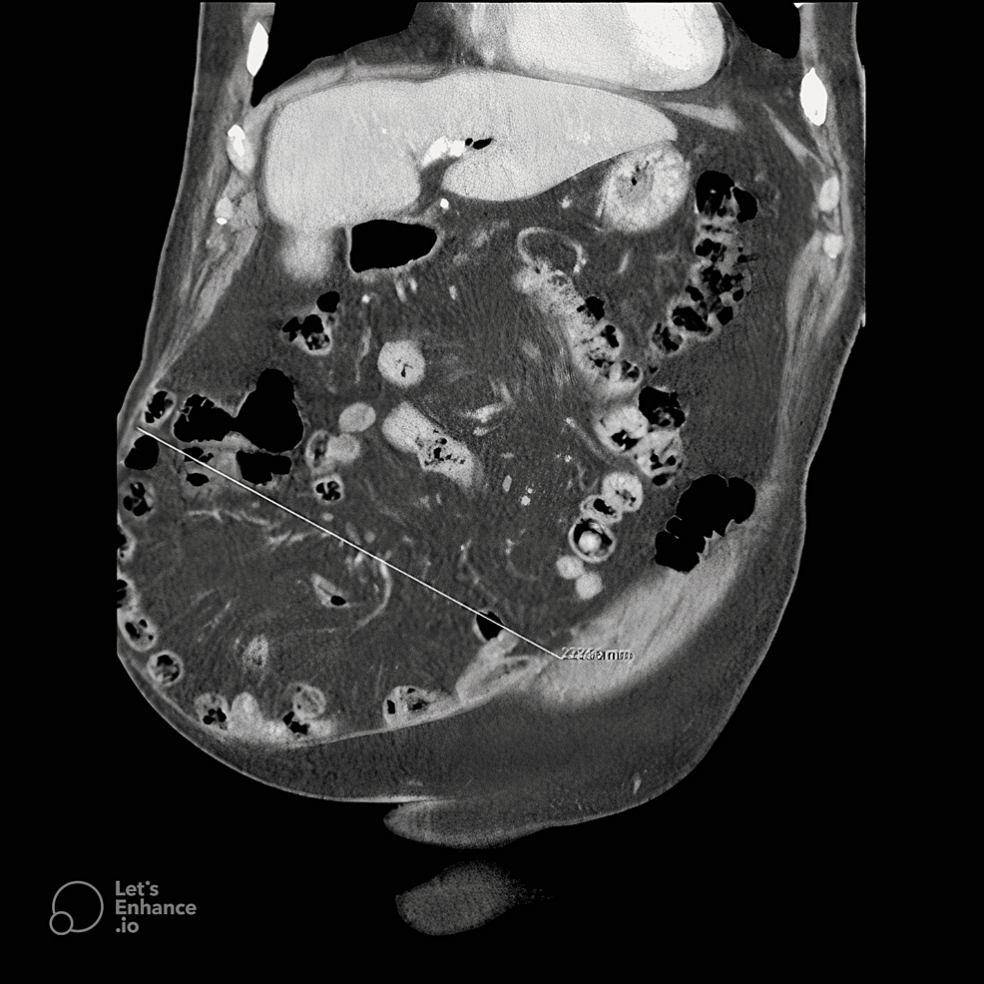
Coronal section CT image of the patient with approximately 24 cm fascial defect

**Figure 3:**
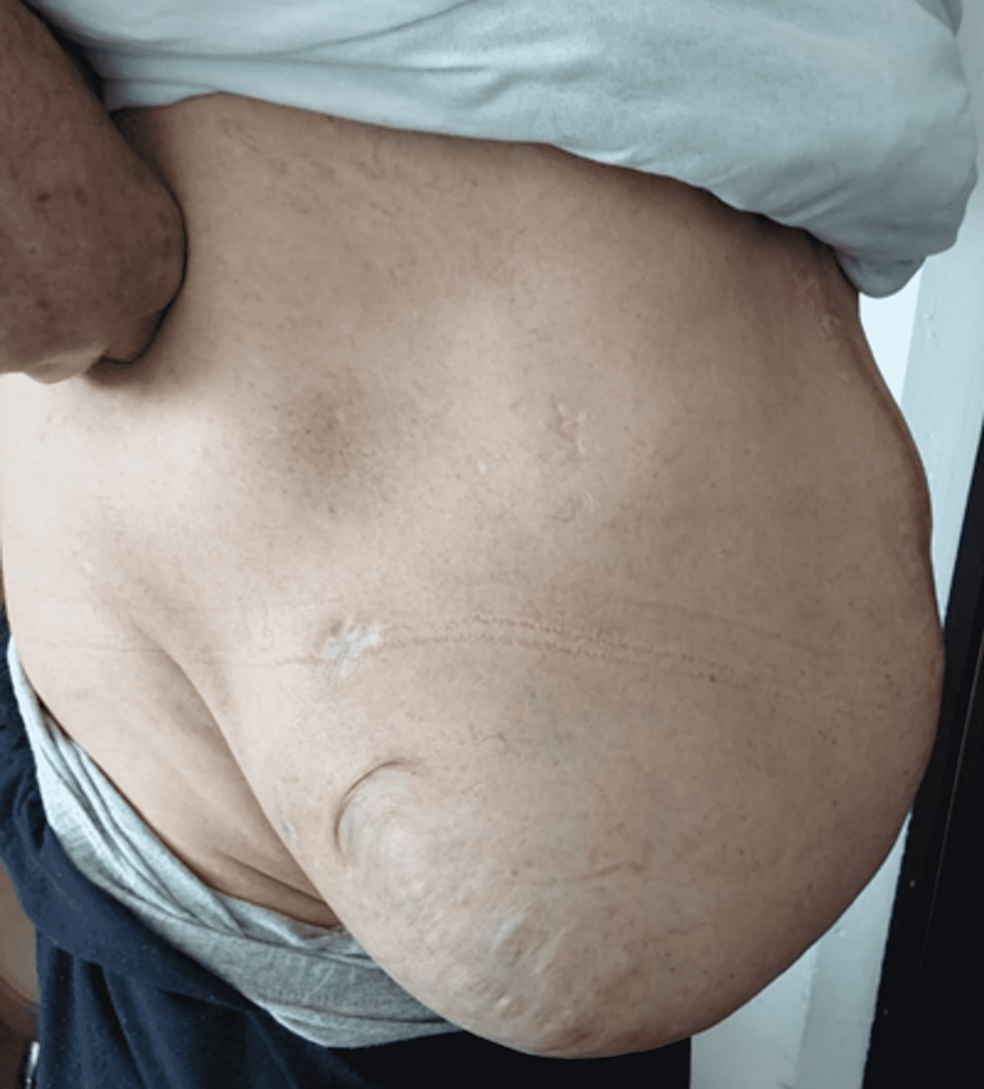
Preoperative standing posture abdomen photograph of the sample patient

**Figure 4:**
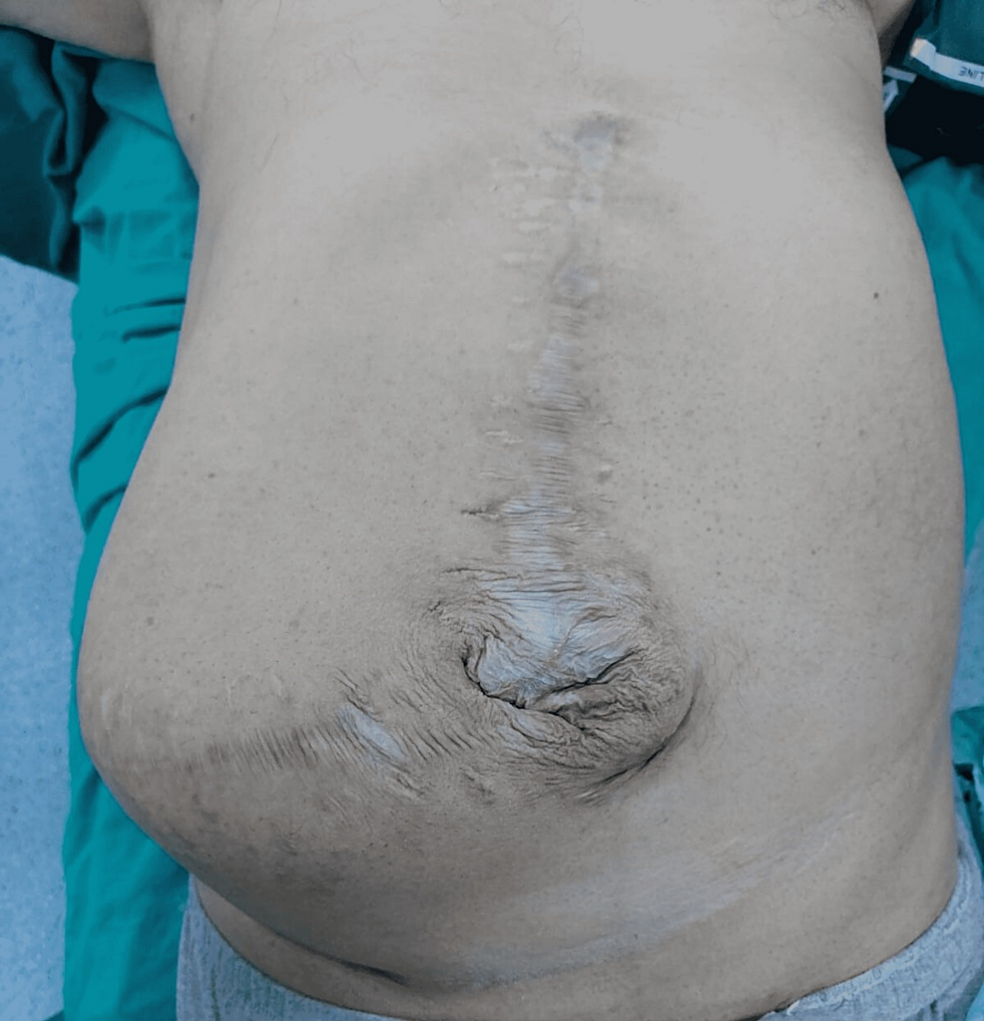
Abdominal photograph of the sample patient in the supine position on the operating table

Figure 1 and Figure 2 show the axial and coronal sections of the CT images of a sample patient, respectively. Abdominal photographs of the same patient standing up and on the operating table are shown in Figure 3 and Figure 4.

**Figure 5:**
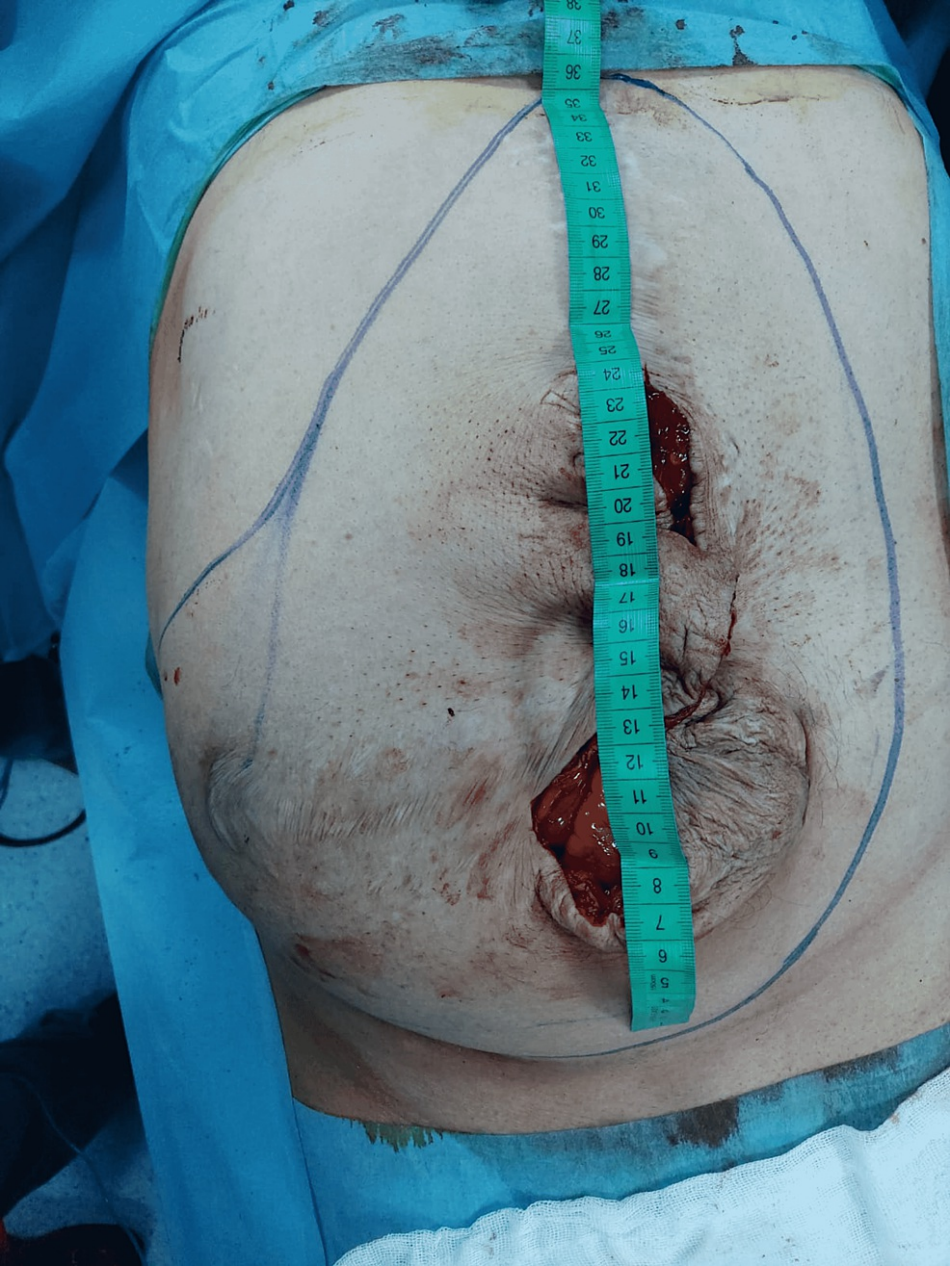
Measurement of the fascial defect and drawing of the intact fascial margins of the sample patient during the operation

**Figure 6:**
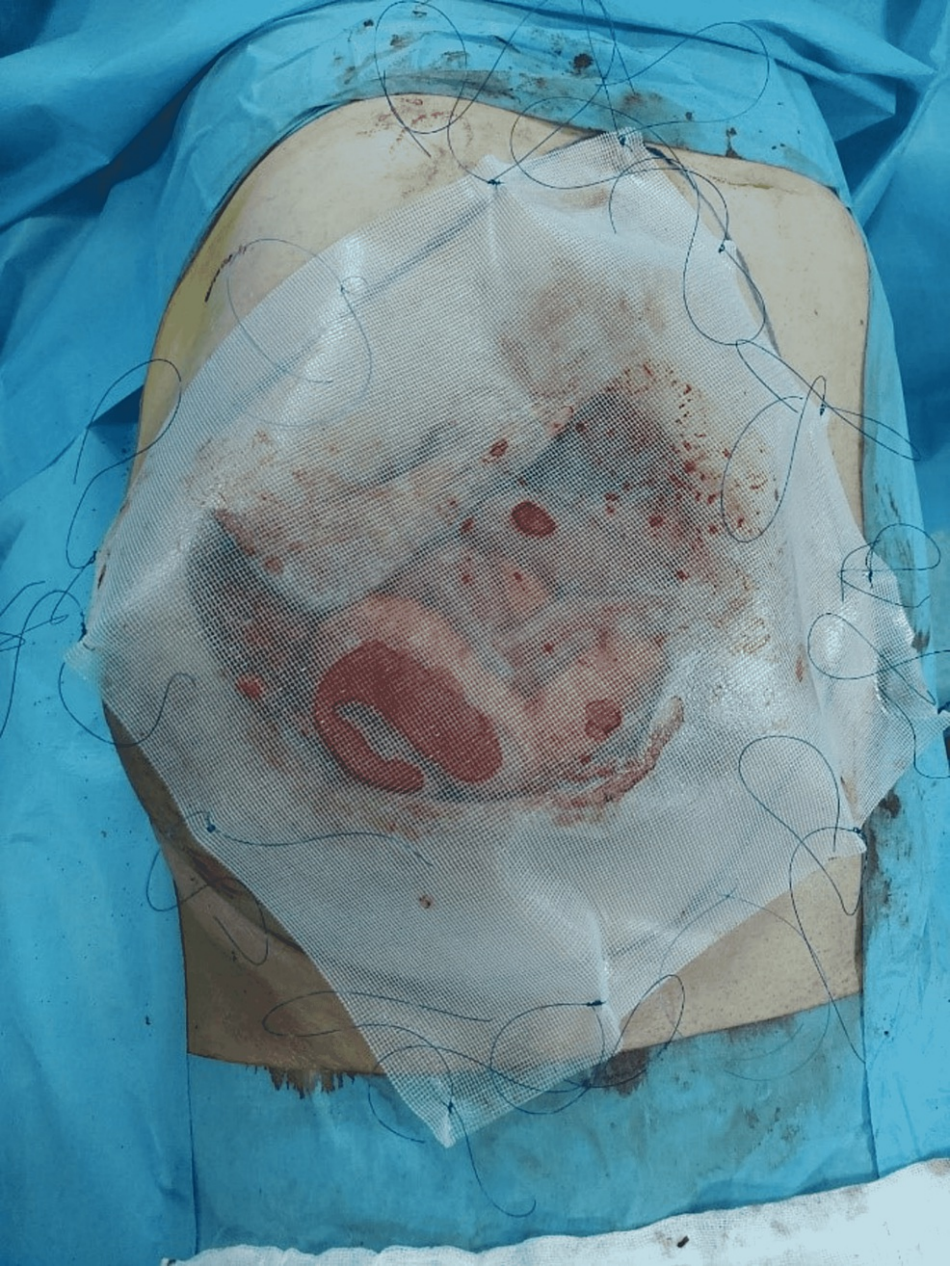
Approximate marking of the fixation points of the prolene sutures attached to the mesh to the fascia.

The skin over the incisional hernia was opened 8-15 cm. Intestinal and omental adhesions in the hernia sac and on the intact fascia surface were removed. The hernia defect was measured and an appropriate-sized dual mesh was prepared (Figures 5-6). The size of the intraperitoneal mesh for hernia margins must be at least 3-5 cm larger than the fascial defect. A small margin may result in insufficient fixation, leading to mesh separation.

In Figure 6, about 16 pieces 2-0 prolene sutures with a length of 15-20 cm are seen for a mesh with the size of 30 cm. Prolene sutures are knotted to the mesh at approximately 4-5 cm intervals from 0.5-1 cm edges of the mesh.

**Figure 7:**
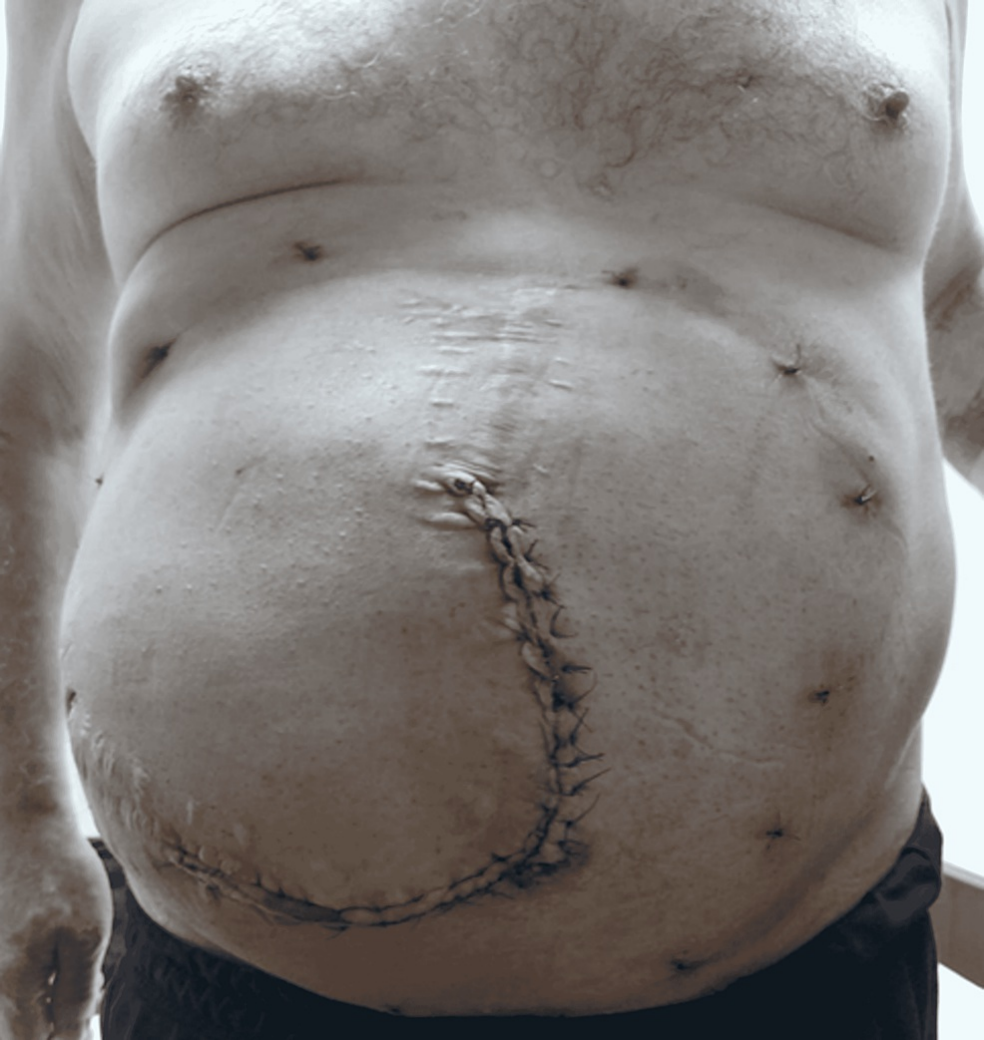
Photograph of the patient on the 15th day after surgery

Prolene sutures will provide a strong fixation of the mesh to the intact fascia (Figure 6). The corresponding points of the sutures on the skin on the abdominal wall were marked with a pencil. The mesh was spread in the abdomen in accordance with the determined projection. Points marked with a pencil on the skin were opened approximately 2 mm with a wedge-tipped scalpel (Scalpel No. 11). Once all prolene sutures were pulled out of the abdomen with an easy close thread-holding trocar from the areas opened with a scalpel, the knots were tied under the skin (Figure 7). A 0.5 cm incision was made in the middle of the mesh and the mesh was stapled with a 5 mm laparoscopic absorbable tacker stapler. The mesh margins were stapled to the abdominal wall with a tacker between the prolene sutures. Refixation with the tacker was visualized and controlled manually at 2 cm intervals. Refixation was performed to prevent bowel and omental herniation.

The incision on the hernia and the places where the transfacial prolene sutures passed and embedded under the skin were sutured. The intermittent circumferential sutures in Figure 7 show the places where the transfacial prolene sutures are knotted under the skin (about 16 pieces).

## Results

Eleven of the patients were male and 14 were female. The mean age is 62±13 years (29-82 years). Average BMI was 32 kg/m2 (20-52 kg/m2). The mean size of the fascial defect was 22±5.5 cm (15-30 cm). The mean operation time was 90 minutes (70-130 minutes) (Table 1). No major intraoperative complications were observed in any of the patients. Postoperative minor complications were seen in six patients according to the Clavien dindo classification [13]. Superficial surgical site infection developed in two patients, which healed with antibiotics and wound care. Bleeding from the thread holding trocar passages occurred in one patient on the second day due to the low molecular weight heparin. Temporary ileus was observed in two patients. Hernia recurrence and intestinal fistula were not observed during an average follow-up of 36 months (6-70 months) (Table 2).

**Table 1:** Demographic and perioperative data

Number of patients male / female	11 / 14
Age	62±13 (29-82)
BMI	32 kg / m^2^ (20-52)
Defect diameter	22±5.5 cm (15-30)
Type of anesthesia : general anesthesia / locoregional anesthesia	15 (60%) / 11 (40%) patients
Average Incision length	12 cm (8-15)
Drain application	15 patients (60%)
Average drain stay	4.5 days (4-6)
Average duration of surgery	90 min (70-130)
Average hospital stay	4.5 days (3-5)
Patients with failed previous repair	1 ( 4% )
Mean follow-up duration	36 months (6-70)

**Table 2:** Number of patients with postoperative complications

Complications	n
Superficial skin infection	2
Skin erosion	1
Bleeding	1
ileus due to intestinal adhesion	2

## Discussion

In our study, during an average of 36 months (6-70 months) follow-up, none of the patients developed obstructions or fistulas that required surgical intervention due to the use of dual mesh. In some publications, it has been stated that the use of intraperitoneal mesh increases the rate of intestinal obstruction and fistula [14,15].

Temporary ileus due to intestinal adhesion developed in two patients who healed without the need for surgical intervention. In the literature, the recurrence rate of incisional hernia surgery has been reported as 9-12% [3,14]. Recurrence after incisional hernia may develop due to improper placement of the mesh, inadequate fixation, or the structural properties of the mesh. Recurrences usually occur within the first year, which increases as the follow-up period gets longer. Obesity and midline defect size are risk factors in the development of recurrence in incisional hernia repair [5,15].

In our patient series, the average BMI index of the patients was 32 kg/m2 (20-52 kg/m2) and most of them were in the obese patient group. Again, the size of the defect was 22±5.5 (15-30) cm, and some of the patients were in the group defined as giant incisional hernia with a large fascial defect involving the entire abdominal wall (Figures 3-4). In our series of 25 patients, recurrence was not observed in any patient. The most important reason for this was thought to be the adequate fixation of the mesh to the intact fascia with transfascial prolene sutures (the number of which is determined according to the size of the defect and mesh) of the margins of the mesh.

In the open onlay mesh application method, extensive dissection of the skin with intact fascia is required for laying the mesh. Seroma, bleeding, infection, and skin necrosis are more common with this method. Seroma and hematoma develop in 17-32% of the patients, therefore, drainage is recommended for patients [11]. Since the fascia and skin are not detached in our method, the risk of postoperative seroma, bleeding, infection and skin necrosis are reduced. Again, since the fascia is not widely detached, the need for the use of painkillers after surgery is reduced. By providing early mobilization of the patients, postoperative deep vein thrombosis and respiratory problems due to the surgical operation were not observed (Tables 1-2).

Our study has some limitations as it is retrospective and includes a small group of patients. Therefore, the analysis may be prone to selection bias. To achieve more accurate results, prospective, randomized clinical and technical studies with similar patient groups are required. Incisional hernia using intraperitoneal dual mesh will need to be investigated for long-term outcomes in terms of hernia recurrence. More research and longer patient follow-up are needed to understand the effect of using intraperitoneal dual mesh on adhesion and fistula development. The follow-up period was limited and therefore the generalizability of the findings will need to be confirmed by longer follow-up periods with extended uses.

## Conclusions

In this study, it was concluded that in the treatment of giant incisional hernias of the abdominal wall, dual meshes applied intraperitoneally with the open method can be used safely, with low complication and recurrence rates and should be kept in mind in the selection of treatment. There were no serious complications requiring intervention due to the use of dual intraperitoneal mesh. In addition, it is thought that conducting long-term randomized controlled studies will contribute more to the literature.

## References

[1] Fink C, Baumann P, Wente MN (2014). Incisional hernia rate 3 years after midline laparotomy. Br J Surg.

[2] Eker HH, Hansson BM, Buunen M (2013). Laparoscopic vs. open incisional hernia repair: a randomized clinical trial. JAMA Surg.

[3] Eriksson A, Rosenberg J, Bisgaard T (2014). Surgical treatment for giant incisional hernia: a qualitative systematic review. Hernia.

[4] Vorst AL, Kaoutzanis C, Carbonell AM, Franz MG (2015). Evolution and advances in laparoscopic ventral and incisional hernia repair. World J Gastrointest Surg.

[5] Parker SG, Mallett S, Quinn L (2021). Identifying predictors of ventral hernia recurrence: systematic review and meta-analysis. BJS Open.

[6] Kulaçoğlu H (2015). Current options in umbilical hernia repair in adult patients. Ulus Cerrahi Derg.

[7] Kaufmann R, Halm JA, Eker HH (2018). Mesh versus suture repair of umbilical hernia in adults: a randomised, double-blind, controlled, multicentre trial. Lancet.

[8] Montgomery A (2013). The battle between biological and synthetic meshes in ventral hernia repair. Hernia.

[9] Shankaran V, Weber DJ, Reed RL 2nd, Luchette FA (2011). A review of available prosthetics for ventral hernia repair. Ann Surg.

[10] Gönüllü D, Nihat Köksoy F (2015). Fıtık tamirinde kullanılan prostetik materyaller [The prosthetic materials in hernia repair]. J Acad Res Med.

[11] Alimi Y, Merle C, Sosin M, Mahan M, Bhanot P (2020202022). Mesh and plane selection: a summary of options and outcomes. Plast Aesthetic Res.

[12] Cobb WS (2018). A current review of synthetic meshes in abdominal wall reconstruction. Plast Reconstr Surg.

[13] Dindo D, Demartines N, Clavien PA (2004). Classification of surgical complications: a new proposal with evaluation in a cohort of 6336 patients and results of a survey. Ann Surg.

[14] Arnold MR, Kao AM, Otero J (2020). Mesh fistula after ventral hernia repair: what is the optimal management?. Surgery.

[15] Suwa K, Okamoto T, Yanaga K (2018). Is fascial defect closure with intraperitoneal onlay mesh superior to standard intraperitoneal onlay mesh for laparoscopic repair of large incisional hernia?. Asian J Endosc Surg.

